# Prevalence and risk factors for diabetic retinopathy at diagnosis of type 2 diabetes: an observational study of 77 681 patients from the Swedish National Diabetes Registry

**DOI:** 10.1136/bmjdrc-2023-003976

**Published:** 2024-06-04

**Authors:** Sheyda Sofizadeh, Katarina Eeg-Olofsson, Marcus Lind

**Affiliations:** 1 Department of Medicine, NU-Hospital Group, Uddevalla, Sweden; 2 Department of Molecular and Clinical Medicine, University of Gothenburg, Gothenburg, Sweden; 3 National Diabetes Register, Centre of Registers, Gothenburg, Sweden; 4 Department of Medicine, Sahlgrenska University Hospital, Gothenburg, Sweden

**Keywords:** Diabetes Mellitus, Type 2, Diabetic Retinopathy, Prediabetic State, Glycated Hemoglobin A

## Abstract

**Introduction:**

To assess the prevalence of diabetic retinopathy (DR) in persons with newly diagnosed type 2 diabetes (T2D) to understand the potential need for intensified screening for early detection of T2D.

**Research design and methods:**

Individuals from the Swedish National Diabetes Registry with a retinal photo <2 years after diagnosis of T2D were included. The proportion of patients with retinopathy (simplex or worse) was assessed. Patient characteristics and risk factors at diagnosis were analyzed in relation to DR with logistic regression.

**Results:**

In total, 77 681 individuals with newly diagnosed T2D, mean age 62.6 years, 41.1% females were included. Of these, 13 329 (17.2%) had DR.

DR was more common in older persons (adjusted OR 1.03 per 10-year increase, 95% CI 1.01 to 1.05) and men compared with women, OR 1.10 (1.05 to 1.14). Other variables associated with DR were OR (95% CI): lower education 1.08 (1.02 to 1.14); previous stroke 1.18 (1.07 to 1.30); chronic kidney disease 1.29 (1.07 to 1.56); treatment with acetylsalicylic acid 1.14 (1.07 to 1.21); ACE inhibitors 1.12 (1.05 to 1.19); and alpha blockers 1.41 (1.15 to 1.73). DR was more common in individuals born in Asia (OR 1.16, 95% CI 1.08 to 1.25) and European countries other than those born in Sweden (OR 1.11, 95% CI 1.05 to 1.18).

**Conclusions:**

Intensified focus on screening of T2D may be needed in Sweden in clinical practice since nearly one-fifth of persons have retinopathy at diagnosis of T2D. The prevalence of DR was higher in men, birthplace outside of Sweden, and those with a history of stroke, kidney disease, and hypertension.

**WHAT IS ALREADY KNOWN ON THIS TOPIC**

- Diabetic retinopathy at diagnosis of type 2 diabetes (T2D) is used as a surrogate marker to indicate late detected T2D, but contemporary and population-based studies are sparse.

**WHAT THIS STUDY ADDS**

- The study reveals that a significant proportion (17.2%) of individuals newly diagnosed with T2D in Sweden already have DR at diagnosis, indicating that a significant proportion of patients have had long-term hyperglycemia before diagnosis.

**HOW THIS STUDY MIGHT AFFECT RESEARCH, PRACTICE, OR POLICY**

- Attention is needed in clinical practice in Sweden regarding screening for T2D in persons with a risk profile and further research is urgently needed regarding potential benefits of structured screening in the population.

## Introduction

 Diabetic retinopathy (DR) is the most common microvascular complication of diabetes.^1^ High blood glucose levels are a critical risk factor for DR, and the risk and severity of DR are directly related to glycated hemoglobin A1c (HbA1c) level over time in both type 1 diabetes and type 2 diabetes (T2D).^2^,^5^ Since DR typically develops over several years, individuals with DR at diagnosis of T2D generally have elevated blood glucose levels long before diagnosis.^6^ Hypertension in conjunction with hyperglycemia is also a well-established risk factor for DR progression.^7^ Other risk factors that have been associated with retinopathy in persons with T2D are Body Mass Index (BMI), dyslipidemia, insulin treatment, and nephropathy.^8^,^11^

The Swedish National Diabetes Registry (NDR) includes the majority of persons with T2D within the country.^12^ Diabetes care in Sweden has significantly improved over time and more patients are reaching glucose control targets. Given intensive treatment in patients with newly diagnosed T2D, undetected hyperglycemia before diagnosis of T2D may be at least as harmful or more so than after diagnosis of T2D. When T2D is undetected individuals may unknowingly have glycemic levels clearly above targets, while after diagnosis modern diabetes care enables patients in many instances to achieve HbA1c targets associated with low risk of diabetes complications.^12^ Early hyperglycemia can also be detrimental over time by virtue of legacy effects, and before diagnosis patients do not receive the same level of attention in terms of screening and treatment for complications.^13 14^

Prevalence of retinopathy at diagnosis of T2D has been used as a surrogate marker for late detected T2D in several other studies.^15^ The aim of the current study was to evaluate to what extent DR exists in persons with newly diagnosed T2D in Sweden and to investigate factors related to increased risk of DR among patients included in the NDR, which includes the absolute majority of persons with T2D in Sweden.

## Research design and methods

The study was approved by the Swedish Ethical Review Authority (Dnr 977-17).

### Data sources

We conducted a registry-based study using data from the NDR. After patients provide verbal informed consent, data are reported directly to the NDR from clinical visits to primary care clinics and hospital diabetes clinics^12 16^ and include risk factors, medications, and complications for individuals with diabetes. Data for the current population of persons with T2D were linked with data from the Swedish Cause of Death Registry, the National Inpatient and Outpatient Registries, the Prescribed Drug Registry, and the Longitudinal Integration Database for Health Insurance and Labour Market Studies.^16^,^18^

### Study population

Individuals diagnosed with T2D from January 1, 2015 to December 31, 2019 with data about DR less than 2 years after diagnosis of T2D were included. Retinal screening is recommended to be performed soon after diagnosis of T2D. Retinal screening is performed by an ophthalmologist or a nurse specialized in ophthalmology. If more severe stages of retinopathy exist, an ophthalmologist is consulted. Information on retinopathy is recorded in the NDR by nurses and physicians working in primary care and outpatient diabetes clinics at hospital. Retinopathy is recorded as non, simplex, non-proliferative, or proliferative retinopathy. However, the variable with best coverage only includes information on whether any retinopathy exists. This variable was used in the current study since the prevalence of DR at diagnosis of T2D was estimated. The procedure for retinal screening has been described in greater detail in earlier studies.^19^

T2D diagnosis required a clinical diagnosis of T2D and fulfilling the following epidemiologic definition: treatment with either diet or non-insulin antihyperglycemic agents only or diagnosis at 40 years of age or older receiving insulin therapy or insulin and oral antihyperglycemic agents.^16 20^ Persons with a diagnosis of type 1 diabetes or less than 18 years of age at index date were excluded.

**Figure 1:**
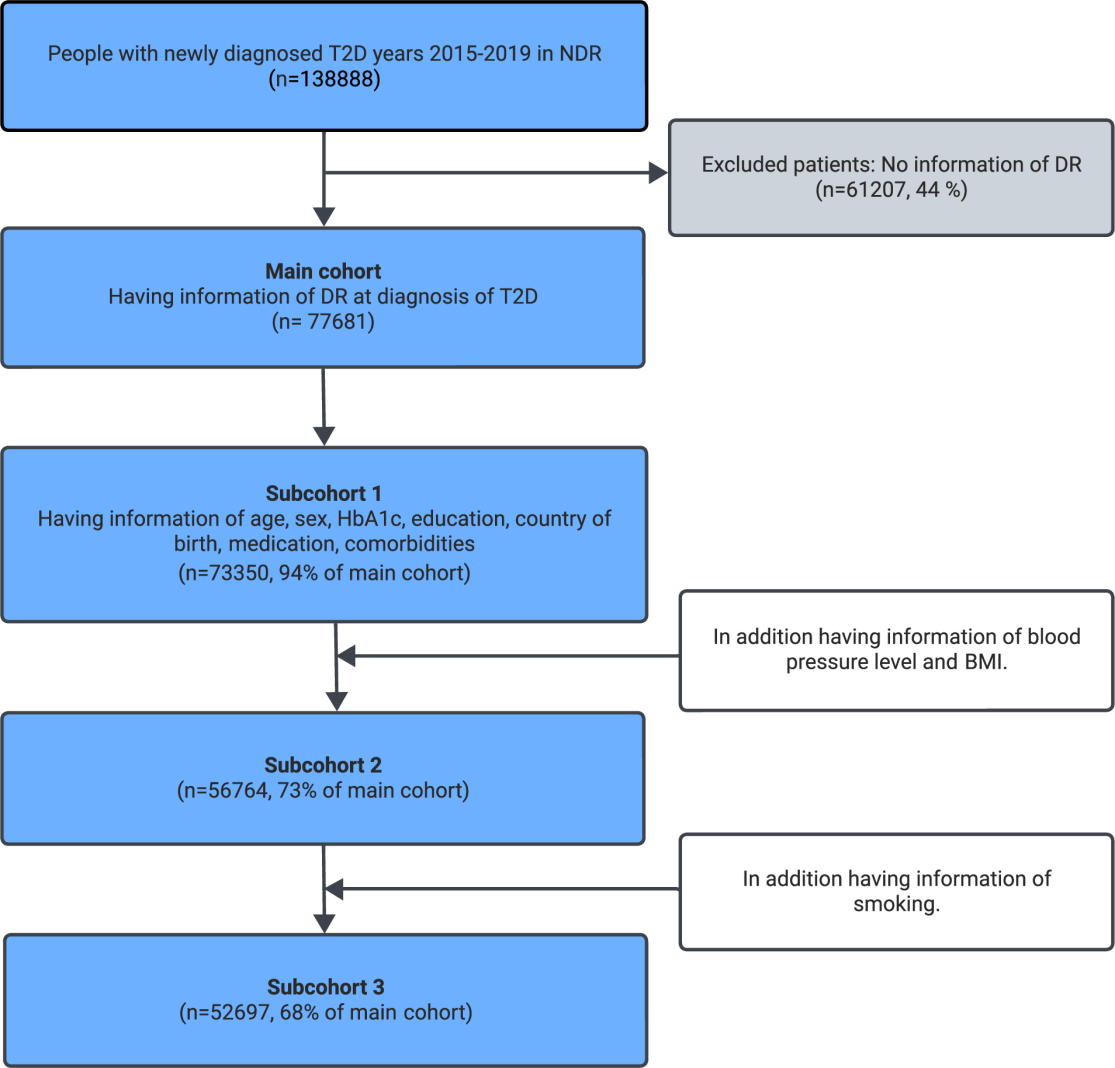
Flow chart of participants in the current study recruited from the National Diabetes Registry (NDR). BMI, Body Mass Index; DR, diabetic retinopathy; HbA1c, glycated hemoglobin A1c; T2D, type 2 diabetes.

There were 138 888 adults in the NDR with newly diagnosed T2D. Of these, 61 207 (44%) did not have data about DR less than 2 years after diagnosis (figure 1). A total of 77 681 persons with T2D remained and were included in the cohort.

### Study procedures

The number and proportion of patients with DR at diagnosis of T2D were calculated. The following variables were evaluated if they were related to the presence of DR: age, sex, smoking, HbA1c level, BMI, blood pressure, level of education, geographic area of birth, diabetes-related medications, renal complications, and cardiovascular comorbidities.

To be representative of the time point of T2D diagnosis (first entry date in the NDR at year of diagnosis), HbA1c and blood pressure measurements had to exist less than 4 weeks after diagnosis of diabetes to be included in the analyses. BMI and smoking data had to exist within 6 weeks and 6 months after diagnosis, respectively.

HbA1c was reported according to the International Federation of Clinical Chemistry standard, measured in mmol/mol, and converted to percent units according to the National Glycohemoglobin Standardization Program for dual reporting criteria.^21^ Laboratory methods at participating care units for analyzing HbA1c were regularly checked with central reference samples of HbA1c to ensure high accuracy.^22^ HbA1c categories included commonly used targets of HbA1c as well as cut-offs used for very poor glucose control in the NDR of 70 mmol/mol (8.6%).^12 23^ HbA1c was categorized as <48 mmol/mol (6.5%), 48–52 mmol/mol (6.5%–6.9%), 53–57 mmol/mol (7.0%–7.4%), 58–70 mmol/mol (7.5%–8.6%), and >70 mmol/mol (>8.6%). Blood pressure was defined as the mean value of two supine readings with a cuff of appropriate size and after at least 5 min of rest. Systolic blood pressure (SBP) was categorized as <110 mm Hg with increments of 10 mm Hg with the highest category ≥140 mm Hg, diastolic blood pressure (DBP) as <60, 60–<70, 70–<80, 80–<85, and ≥ 85 mm Hg. Commonly used levels of BMI for classifying underweight normal weight, obesity, and severe obesity were used when evaluating BMI^23 24^: <18.5, 18.5–<25, 25–<30, 30–<35, and ≥35 kg/m^2^. Smoking was categorized as “No” (never smokers and previous smokers) versus “Yes” (current smokers), education level as up to 9 years, 10–12 years, or college/university; and geographic area of birth as Africa, Asia, Europe (excluding Sweden), Oceania, North America, South America, and Sweden.

International classification of diseases (ICD-10) codes were used to define study comorbidities (ICD codes are described in online supplemental material). Comorbidities were investigated back until year 1997 when ICD-10 was introduced. If a diagnosis of a certain comorbidity existed during the time period from 1997 until diagnosis of T2D, it was regarded as prevalent. The following comorbidities were evaluated: coronary heart disease (CHD), stroke, atrial fibrillation, heart failure, coronary artery bypass graft, peripheral arterial disease and chronic kidney disease (CKD).

Anatomical therapeutic classifications based on the prescribed drug registry were used for evaluation of drugs. The following classes of drugs were evaluated: acetylsalicylic acid, antihypertensive, beta-blockers, ACE inhibitors, angiotensin II receptor blockers, calcium channel blockers, alpha-blockers, and diuretics. Presence of hyperlipidemia and hypertension before diagnosis of T2D was both defined by use of prescribed drugs before diagnosis of T2D.

### Statistical analyses

Descriptive statistics are presented for patients with T2D who had a registration regarding retinopathy in NDR <2 years after diagnosis of T2D (Main cohort) and patients with T2D without registration about retinopathy examinations in the same time window (Excluded group). The groups were compared with comparative tests (t-test and standard mean difference) to describe any differences.^25^,^27^

In patients with newly diagnosed T2D with a registration of retinopathy examination (Main cohort), characteristics of the proportion of patients with DR were compared with those without DR and descriptive statistics are presented and the groups were compared with t-test and χ^2^-test. Multiple logistic regression was used to evaluate variables associated with DR at diagnosis of T2D. Results are presented as adjusted ORs with 95% CIs.

Variables associated with DR were analyzed with logistic regression in three different subcohorts to include as many individuals as possible for a certain variable. Missing variables were handled in the regression analysis using complete cases. Each analysis included individuals with data on all variables. Subcohort 1 (n=73 350) included all patients with data on age, sex, educational level, comorbidities, diabetes-related drugs, and HbA1c. Subcohort 2 (n=56 764) included patients who had blood pressure and BMI data in addition to subcohort 1. Subcohort 3 (n=52 697) included patients who had smoking data in addition to subcohort 2. Two-tailed tests were used and a significance level of 0.05 was applied. The results are reported as OR with their 95% CI. SAS V.9.4 was used for statistical analyses.

### Role of the funding source

The funders had no role in study design, data collection and analysis, preparation of the manuscript, or decision to submit for publication.

## Results

### Prevalence of DR

A total of 77 681 individuals with newly diagnosed T2D were included in the current study (main cohort). Overall, patient characteristics were numerically similar to the 61 207 patients without information on retinopathy less than 2 years after diagnosis of T2D (table 1). Mean age in the main cohort compared with excluded patients was 62.6 and 62.8 years and 41.1% and 41.8% were females, respectively. Geographic area of birth was also similar with 76.2% and 74.2% born in Sweden, 11.6% and 11.7% in other European countries, and 8.5% vs 9.7% in Asia, respectively. Education level 10–12 years was 48.1% vs 46.1% and college/university was 22.9% vs 22.5%, respectively.

**Table 1:** Patient characteristics at diagnosis of type 2 diabetes (T2D) shown both for the main cohort and excluded persons without information of retinopathy screening within 2 years of their diagnosis

	Main cohort (n=77 681)	Excluded (n=61 207)	P value	SMD
Age years, mean (SD)	62.6 (12.4)	62.8 (13.7)	0.012	0.014
Sex female, n (%)	31 944 (41.1)	25 593 (41.8)	0.010	0.014
Smoking, n (%)				
No smoking	58 944 (84.7)	38 006 (83.7)	0.001	0.020
Smoking	10 622 (15.3)	7423 (16.3)		
HbA1c (mmol/mol) at inclusion, mean (SD)	58.1 (20.9)	54.3 (18.2)	<0.001	0.194
HbA1c (mmol/mol) at inclusion, n (%)				
<48	26 726 (36.0)	24 736 (43.2)	<0.001	0.195
48–52	15 852 (21.3)	12 293 (21.5)		
53–57	7885 (10.6)	6059 (10.6)		
58–70	9305 (12.5)	6611 (11.5)		
>70	14 558 (19.6)	7569 (13.2)		
Place of birth, n (%)				
Sweden	59 169 (76.2)	45 384 (74.2)	<0.001	0.058
Europe except Sweden	9014 (11.6)	7149 (11.7)		
North America	205 (0.3)	200 (0.3)		
South America	691 (0.9)	544 (0.9)		
Asia	6589 (8.5)	5931 (9.7)		
Africa	1989 (2.6)	1968 (3.2)		
Oceania	12 (0.0)	14 (0.0)		
Lipid-lowering therapy, n (%)	29 286 (37.7)	24 148 (39.5)	0.006	0.015
Stroke, n (%)	2940 (3.8)	2749 (4.5)	<0.001	0.056
Coronary heart disease, n (%)	9318 (12.0)	8276 (13.5)	<0.001	0.057
Atrial fibrillation, n (%)	6225 (8.0)	5855 (9.6)	<0.001	0.055
Heart failure, n (%)	3592 (4.6)	3644 (6.0)	<0.001	0.059
Peripheral arterial disease, n (%)	692 (0.9)	628 (1.0)	0.011	0.014
Coronary artery bypass graft, n (%)	982 (1.3)	884 (1.4)	0.004	0.016
Chronic kidney disease, n (%)	722 (0.9)	834 (1.4)	<0.001	0.041
Any hypertensive treatment, n (%)	50 208 (64.6)	41 198 (67.3)	<0.001	0.056
Acetylsalicylic acid, n (%)	14 562 (18.7)	12 237 (20.0)	<0.001	0.032
ACE inhibitor, n (%)	19 037 (24.5)	14 864 (24.3)	0.343	0.005
Angiotensin receptor blocker, n (%)	19 971 (25.7)	17 141 (28.0)	<0.001	0.052
Alpha-blocker, n (%)	601 (0.8)	551 (0.9)	0.011	0.014
Beta-blocker, n (%)	25 990 (33.5)	22 049 (36.0)	<0.001	0.054
Calcium channel antagonist/blocker, n (%)	19 942 (25.7)	16 580 (27.1)	<0.001	0.032
Diuretics, n (%)	15 677 (20.2)	13 420 (21.9)	<0.001	0.043
Education category, n (%)				
9 years	19 691 (29.0)	12 675 (31.4)	<0.001	0.054
10–12 years	32 700 (48.1)	18 605 (46.1)		
College/university	15 593 (22.9)	9062 (22.5)		
Systolic blood pressure (mm Hg), mean (SD)	135.9 (16.4)	135.2 (16.5)	<0.001	0.043
Diastolic blood pressure (mm Hg), mean (SD)	80.7 (10.3)	80.2 (10.4)	<0.001	0.048
BMI (kg/m^2^), mean (SD)	31.3 (5.9)	31.2 (6.0)	<0.001	0.027

Excluded persons had no information on DR at the time of diagnosis of T2D in the Swedish National Diabetes Register. P values are obtained from t-tests for continuous variables and χ2-tests for frequencies.

HbA1c, glycated hemoglobin A1c; SMD, standardized mean difference.

The frequency of comorbidities was numerically similar with a prevalence of CHD of 12.0% vs 13.5% and stroke 3.8% vs 4.5%, respectively. Mean HbA1c level was 58.1 mmol/mol (7.5%) and 54.3 mmol/mol (7.1%). Although significant differences between the groups existed for several variables, the numeric differences were small illustrated by low standard mean differences (table 1). Percentage of patients in the main cohort and excluded patients having missing data around the time of T2D diagnosis of HbA1c, smoking, BMI, and blood pressure were overall similar in the two groups, but a slightly larger proportion of included patients had missing data on blood pressure (online supplemental table S1).

In total, 13 329 (17.2%) had DR at diagnosis of T2D. Patient characteristics for persons with and without DR in the main cohort by diagnosis of DR are presented in table 2.

**Table 2:** Patient characteristics main cohort participants with and without diabetic retinopathy (DR) at diagnosis of type 2 diabetes (T2D)

Characteristics	All patients n=77 681 (100%)	With DR at diagnosis of T2D Yes DR n=13 329 (17.2%)	Without DR at diagnosis of T2D No DR n=64 352 (82.8%)	P value
Age years, mean (SD)	62.62 (12.41)	62.6 (12.4)	62.6 (12.4)	0.81
Age category, n (%)				
<55 years	19 560 (25.2)	3432 (25.7)	16 128 (25.1)	0.082
55–64 years	20 623 (26.5)	3540 (26.6)	17 083 (26.5)	
65–74 years	24 587 (31.7)	4103 (30.8)	20 484 (31.8)	
75+ years	12 911 (16.6)	2254 (16.9)	10 657 (16.6)	
Sex female, n (%)	31 944 (41.1)	5127 (38.5)	26 817 (41.7)	<0.001
Smoking, n (%)				
No smoking	58 944 (84.7)	9967 (83.7)	48 977 (84.9)	<0.001
Smoking	10 622 (15.3)	1938 (16.3)	8684 (15.1)	
HbA1c (mmol/mol) at inclusion, mean (SD)	58.07 (20.93)	61.8 (23.12)	57.3 (20.4)	<0.001
HbA1c (mmol/mol) at inclusion, n (%)				
<48	26 726 (36.0)	3886 (30.6)	22 840 (37.1)	<0.001
48–52	15 852 (21.3)	2462 (19.4)	13 390 (21.7)	
53–57	7885 (10.6)	1295 (10.2)	6590 (10.7)	
58–70	9305 (12.5)	1777 (14.0)	7528 (12.2)	
>70	14 558 (19.6)	3278 (25.8)	11 280 (18.3)	
Place of birth, n (%)				
Sweden	59 169 (76.2)	9939 (74.6)	49 230 (76.5)	<0.001
Europe except Sweden	9014 (11.6)	1640 (12.3)	7374 (11.5)	
North America	205 (0.3)	35 (0.3)	170 (0.3)	
South America	691 (0.9)	109 (0.8)	582 (0.9)	
Asia	6589 (8.5)	1253 (9.4)	5336 (8.3)	
Africa	1989 (2.6)	350 (2.6)	1639 (2.5)	
Oceania	12 (0.0)	1 (0.0)	11 (0.0)	
Lipid-lowering therapy, n (%)	29 286 (37.7)	5071 (38.0)	24 215 (37.6)	0.826
Stroke, n (%)	2940 (3.8)	597 (4.5)	2343 (3.6)	<0.001
Coronary heart disease, n (%)	9318 (12.0)	1640 (12.3)	7678 (11.9)	0.006
Atrial fibrillation, n (%)	6225 (8.0)	1099 (8.2)	5126 (8.0)	0.287
Heart failure, n (%)	3592 (4.6)	652 (4.9)	2940 (4.6)	0.111
Peripheral arterial disease, n (%)	692 (0.9)	147 (1.1)	545 (0.8)	0.005
Coronary artery bypass graft, n (%)	982 (1.3)	195 (1.5)	787 (1.2)	0.027
Chronic kidney disease, n (%)	722 (0.9)	158 (1.2)	564 (0.9)	0.001
Any hypertensive treatment, n (%)	50 208 (64.6)	8518 (63.9)	41 690 (64.8)	0.055
Acetylsalicylic acid, n (%)	14 562 (18.7)	2682 (20.1)	11 880 (18.5)	<0.001
ACE inhibitor, n (%)	19 037 (24.5)	3453 (25.9)	15 584 (24.2)	<0.001
Angiotensin receptor blocker, n (%)	19 971 (25.7)	3272 (24.5)	16 699 (25.9)	0.001
Alpha-blocker, n (%)	601 (0.8)	138 (1.0)	463 (0.7)	<0.001
Beta-blocker, n (%)	25 990 (33.5)	4424 (33.2)	21 566 (33.5)	0.480
Calcium channel antagonist/blocker, n (%)	19 942 (25.7)	3495 (26.2)	16 447 (25.6)	0.113
Diuretics, n (%)	15 677 (20.2)	2676 (20.1)	13 001 (20.2)	0.750
Education category, n (%)				
9 years	19 691 (29.0)	3645 (30.7)	16 046 (28.6)	<0.001
10–12 years	32 700 (48.1)	5564 (46.9)	27 136 (48.3)	
College/university	15 593 (22.9)	2648 (22.3)	12 945 (23.1)	
SBP (mm Hg), mean (SD)	135.93 (16.41)	137.5 (17.3)	135.6 (16.20)	<0.001
SBP (mm Hg), n (%)				
<110	1831 (2.6)	302 (2.5)	1529 (2.6)	<0.001
110–<120	5591 (8.0)	841 (7.0)	4750 (8.1)	
120–<130	14 088 (20.0)	2214 (18.5)	11 874 (20.4)	
130–<140	19 201 (27.3)	3112 (26.0)	16 089 (27.6)	
≥140	29 557 (42.1)	5494 (45.9)	24 063 (41.3)	
DBP (mm Hg), mean (SD)	80.71 (10.27)	81.2 (10.63)	80.6 (10.2)	<0.001
DBP (mm Hg), n (%)				
< 60	640 (0.9)	116 (1.0)	524 (0.9)	<0.001
60–<70	5800 (8.3)	974 (8.1)	4826 (8.3)	
70–<80	18 833 (26.8)	3008 (25.2)	15 825 (27.2)	
80–<85	20 560 (29.3)	3481 (29.1)	17 079 (29.3)	
≥85	24 376 (34.7)	4375 (36.6)	20 001 (34.3)	
BMI (kg/m^2^), mean (SD)	31.3 (5.9)	30.9 (5.9)	31.4 (5.9)	<0.001
BMI (kg/m^2^), n (%)				
<18.5	167 (0.3)	38 (0.4)	129 (0.2)	<0.001
18.5–<25	6939 (11.1)	1389 (13.0)	5550 (10.7)	
25–<30	21 861 (34.8)	3817 (35.8)	18 044 (34.6)	
30–<35	19 417 (30.9)	3152 (29.6)	16 265 (31.2)	
≥35	14 373 (22.9)	2264 (21.2)	12 109 (23.2)	

Excluded persons had no information on DR at the time of diagnosis of T2D in the Swedish National Diabetes Register. P values are obtained from t-tests for continuous variables and χ2-tests for frequencies.

BMI, Body Mass Index; DBP, diastolic blood pressure; HbA1c, glycated hemoglobin A1c; SBP, systolic blood pressure;

### Risk factors for DR

We evaluated adjusted ORs in subcohorts of individuals with data on the covariates (figure 1). Characteristics were overall similar in subcohorts 1–3 (online supplemental table S2).

**Figure 2:**
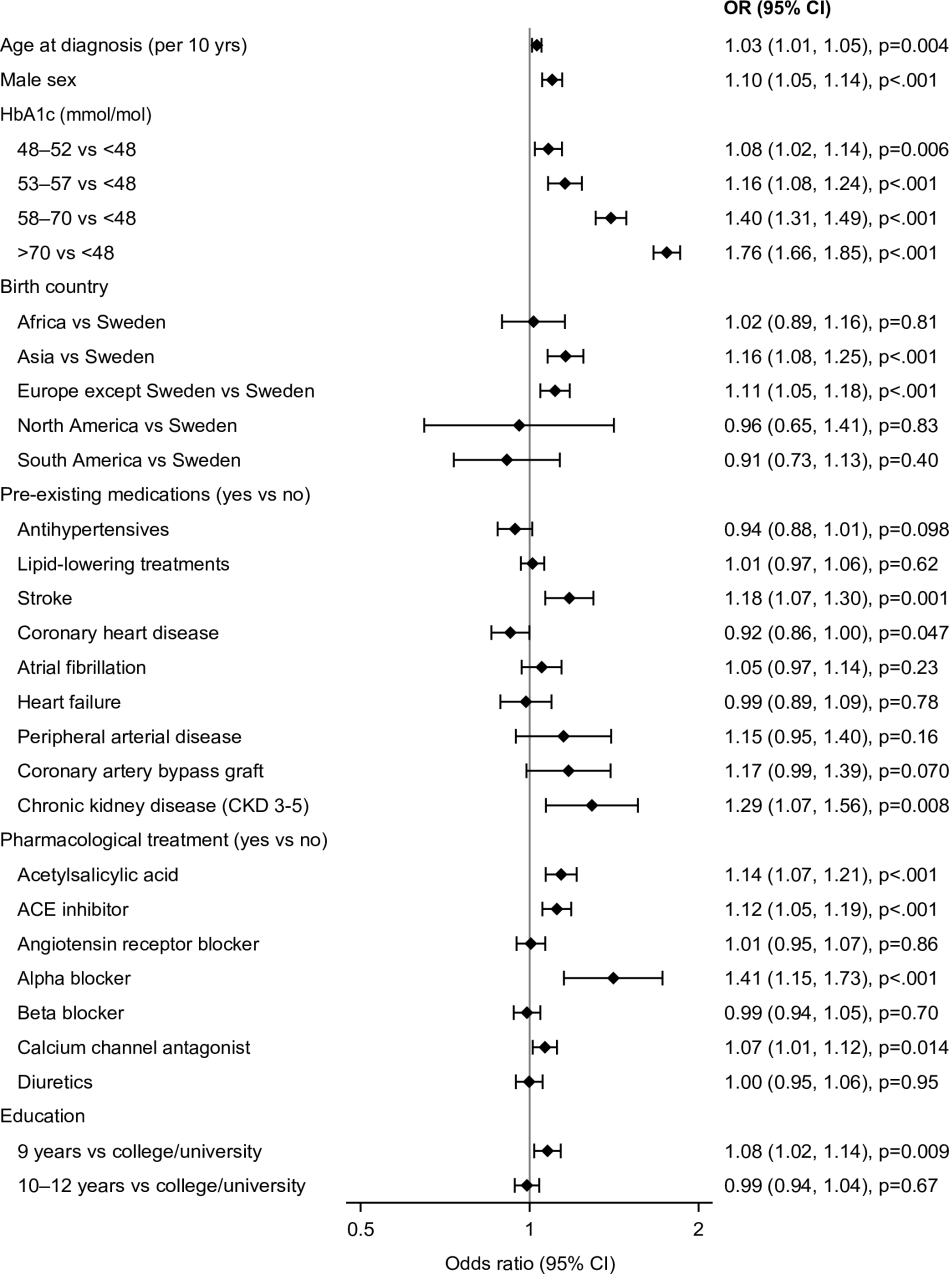
ORs of retinopathy by patient characteristics in persons with newly diagnosed type 2 diabetes from multivariable logistic regression models in subcohort 1 (n=73 350) with information on age, sex, comorbidities, educational level, geographic area of birth, prescribed medications, and glycemic control (HbA1c within 4 weeks after diagnosis). Reference groups for each variable are indicated by the y-axis labels. Points and error bars represent ORs and 95% CIs. HbA1c, glycated hemoglobin A1c.

In subcohort 1, 73 350 (94%) individuals with data on age, sex, comorbidities, educational level, geographic area of birth, prescribed medications, and glycemic control (HbA1c within 4 weeks after diagnosis) were analyzed with logistic regression (figure 2).

DR was more common in older persons, by OR 1.03 (95% CI 1.01 to 1.05, p=0.004) per 10 years increase and more common in men compared with women OR 1.10 (95% CI 1.05 to 1.14, p<0.001). Other variables associated with DR were lower education, OR 1.08 for primary versus college/university (95% CI 1.02 to 1.14, p=0.009), previous stroke, OR 1.18 (95% CI 1.07 to 1.30, p=0.001), CKD, OR 1.29 (95% CI 1.07 to 1.56, p=0.008), treatment with acetylsalicylic acid, OR 1.14 (95% CI 1.07 to 1.21, p<0.001), ACE inhibitors, OR 1.12 (95% CI 1.05 to 1.19, p<0.001), and alpha blockers, OR 1.41 (95% CI 1.15 to 1.73, p<0.001). With respect to geographic area, DR was more common in individuals born in Asia, OR 1.16 (95% CI 1.08 to 1.25, p<0.001) and European countries other than Sweden, OR 1.11 (95% CI 1.05 to 1.18, p<0.001) compared with those born in Sweden (figure 2).

**Figure 3:**
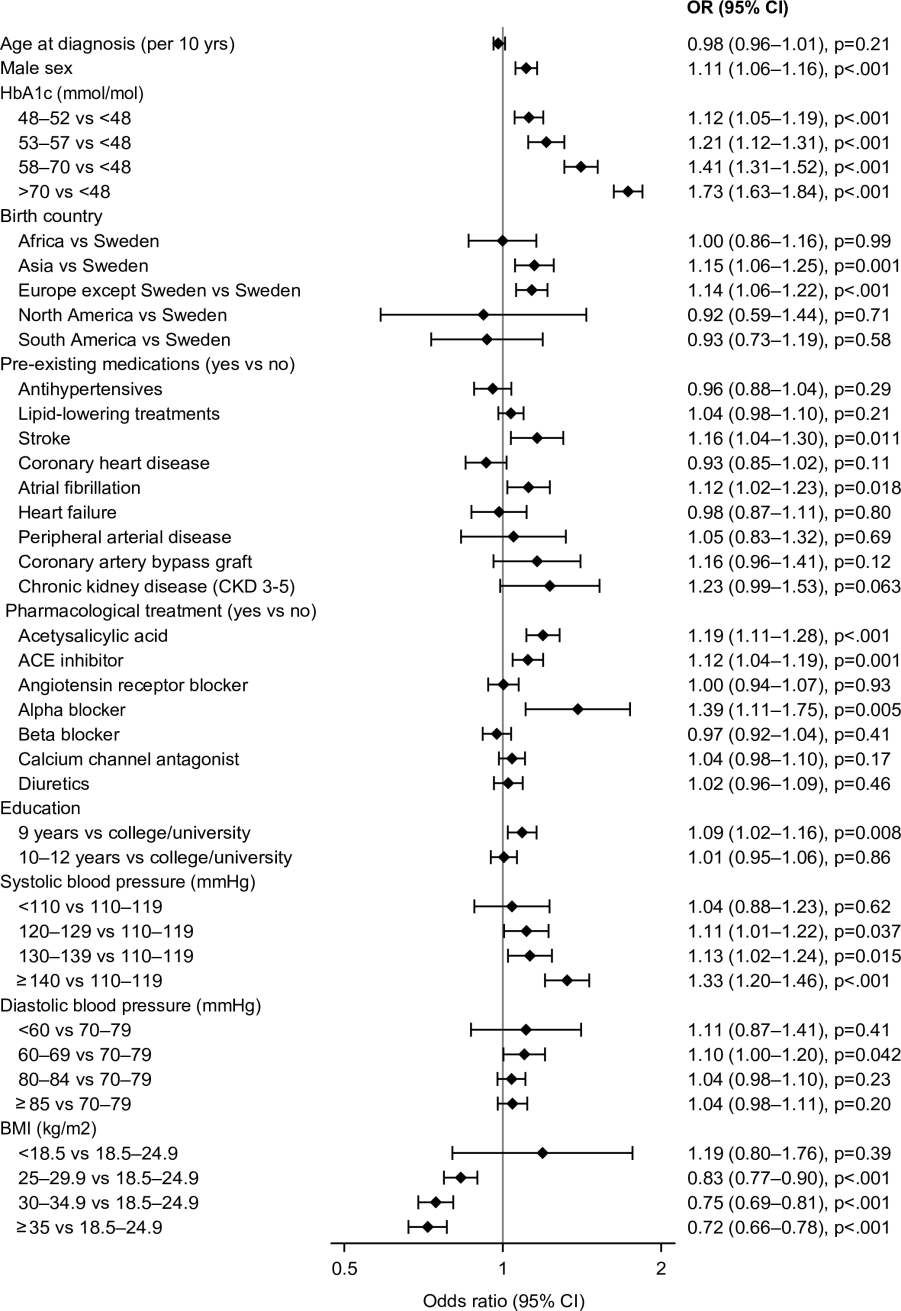
ORs of retinopathy by patient characteristics in persons with newly diagnosed type 2 diabetes (T2D) from multivariable logistic regression models in subcohort 2 (n=56 764) with information on age, sex, comorbidities, educational level, geographic area of birth, prescribed medications, and glycemic control (HbA1c within 4 weeks after diagnosis) and also including systolic blood pressure and diastolic blood pressure less than 4 weeks after diagnosis of T2D and BMI less than 6 weeks after diagnosis. Reference groups for each variable are indicated by the y-axis labels. Points and error bars represent ORs and 95% CIs. BMI, Body Mass Index; HbA1c, glycated hemoglobin A1c.

In subcohort 2, 56 764 patients (73% of the main cohort) additionally had data on SBP and DBP less than 4 weeks after diagnosis of T2D and BMI less than 6 weeks after diagnosis. The risk of DR increased with higher SBP with an OR of 1.33 (95% CI 1.20 to 1.46, p<0.001) for an SBP ≥140 mm Hg compared with those having an SBP of 110–119 mm Hg. In contrast, the risk of DR decreased with higher BMI with an OR of 0.75 (95% CI 0.69 to 0.81, p<0.001) and 0.72 (95% CI 0.66 to 0.78, p<0.001) for those with BMI 30–34.9 and ≥35 kg/m^2^ compared with 18.5–24.9 kg/mg^2^, respectively (figure 3).

In subcohort 3, 52 697 patients (68% in the main cohort) additionally had data on smoking at less than 6 months after diagnosis of T2D. Smoking showed no association with DR with an OR of 1.05 (95% CI 0.95 to 1.12, p=0.17). ORs for other variables were similar to those from subcohort 1 and 2 (data not shown).

## Conclusions

In this nationwide study from Sweden, using DR at diagnosis of T2D as a marker for late detected T2D, almost one-fifth of patients had DR at diagnosis of T2D. DR was more common in men, individuals born in Asia, and those with a history of stroke and kidney disease. High SBP and elevated HbA1c levels were also associated with DR. A higher proportion of patients with normal weight had DR at diagnosis of T2D compared with those who were overweight or obese. DR was less common in individuals with previous CHD.

Prevalence of retinopathy as an indicator for late detected T2D has been used in several earlier studies.^15^ However, contemporary population-based studies of the prevalence of DR are overall spars. In a UK-based study examining newly diagnosed persons with T2D until year 2017, the prevalence of DR ranged from 14% to 25% depending on whether pre-diabetes had been recorded as diagnosis or not before diagnosis of T2D.^28^ A systematic review and meta-analysis including studies generally performed more than 10 years ago found that the pooled prevalence of DR at diagnosis of T2D was 14.6% (95% CI 11.9% to 17.3%).^15^ Some studies have reported that DR is present in up to 15%–20% of patients at the time of diagnosis of T2D, while others have reported that DR is present in around 5%–10%.^615 29^,^33^

Hyperglycemia and hypertension are risk factors for DR in persons with established T2D as confirmed in randomized settings.^2 7^ Studies have also reported hyperglycemia and hypertension to be more common in patients with DR at diagnosis of T2D.^1 15 34^ DR at diagnosis of T2D has also been reported to be more common in persons with renal complications whereas smoking has shown divergent associations.^5 32^ In different populations of individuals with DR has been more common in men compared with women.^35^

Experience from clinical practice and studies in type 1 diabetes, where the initial hyperglycemia is generally more abrupt, suggest that hyperglycemia generally needs to exist over a long period of time before DR appears.^3 4^ Data indicate that diabetes is generally present for at least 5 years before signs of retinopathy appear, and it may be more than 10 years after diagnosis of diabetes before clinical diagnosis of DR.^6^ That almost one-fifth of patients in the current study had DR at diagnosis of T2D indicates that long-standing hyperglycemia before diagnosis of T2D is relatively common in Sweden, and hyperglycemia increases risk of complications at diagnosis of T2D. Furthermore, legacy effects of earlier hyperglycemia may worsen prognosis after diagnosis compared with persons with early detection.^13 14^ Moreover, many individuals do not receive treatments for preventing diabetes complications before diagnosis of T2D such as lipid-lowering and antihypertensive drugs, lifestyle advice, and screening programs for complications.^23^ It is possible that diabetes complications and mortality can be reduced during this high-risk phase if diabetes is detected early, and intensive prevention programs are started. ACE inhibitors and angiotensin-2 receptor blockers are likely beneficial in preventing or slowing the progression of early DR.^36^ Further, studies indicate that the use of antiplatelet/anticoagulant medications may reduce the risk of developing non-proliferative DR among patients with T2D while fibrates may benefit diabetic macular edema.^36 37^

Diabetes care in Sweden has significantly improved over time with a large proportion of persons with T2D obtaining a target HbA1c level <52 mmol/mol (6.9%).^38^ However, that a relatively large proportion of patients have DR at diagnosis of T2D indicates that strategies for detecting T2D at earlier stages need to improve. Although diabetes care for persons with established T2D has substantially improved over time, detecting diabetes at an early stage has not achieved corresponding success.^12^ When clearly elevated glucose levels exist before diagnosis, the harm due to legacy effects will likely not be evident until later years.^13 14^

Guidelines suggest that overweight and obese individuals should be screened for T2D.^23 39^ Other individuals in focus are first-degree relatives of individuals with T2D, that is, having a hereditary component. Specific risk scores exist that can be used for screening for T2D.^40^ However, clearly structured programs for screening risk groups are lacking in most countries, while screening is generally random and, in many instances, may be missed. In the ADDITION study, structured screening for T2D was evaluated, but clear benefits on a population level could not be confirmed.^41^ More research is needed into implementing structured screening programs for at-risk persons with T2D to detect disease at an early stage. Currently, by greater focus in clinical practice by extended screening of T2D, it may also be possible to detect pre-diabetes and prevent T2D more efficiently through lifestyle interventions.^42^

In the current study, most risk factors for DR at diagnosis were expected. However, we did not expect that those with high BMI were less likely to have DR compared with those with normal weight. It is possible that individuals with normal BMI who end up developing T2D may be screened later for T2D after a more long-term hyperglycemia. It was also of interest that individuals born in Asia and then migrating to Sweden had higher risk of DR at diagnosis of T2D compared with those born in Sweden. One possible explanation is that this patient group may be less informed regarding T2D risk factors and need for screening. Another is that disease progression differs since persons born in Asia who are not overweight or obese generally develop T2D more often compared with those born in Western countries.^43 44^ Retinopathy progression has shown to be more common in certain ethnic groups in earlier studies including Indian, Pakistani, and South Asian African ethnic groups.^10 45 46^

One strength of the current study is the population-based design where the NDR covers the majority of persons with T2D in Sweden. A limitation is that 44% of the newly diagnosed had no data available in the NDR on retinopathy less than 2 years after diagnosis of T2D and were therefore not included in the current analysis. However, patient characteristics were similar overall among included and excluded patients indicating major selection bias is not likely. Although some patient characteristics differed between the included and excluded patients, they were overall numerically small, except for HbA1c where a somewhat greater difference existed at 58.1 mmol/mol (7.5%) vs 54.3 mmol/mol (7.1%). Mean HbA1c was somewhat lower among excluded patients possibly indicating slightly lower prevalence of DR in this population. Nevertheless, even if a lower proportion of excluded patients had DR, the overall proportion of patients having DR would still be relatively high. It is unclear to what extent those patients without data on DR in the NDR lacked a retinal screening or if results of screening had not been recorded. The NDR is dependent on health professionals registering information on retinopathy in the NDR based on clinical eye examinations. The study was limited that a minority of patients had information on albuminuria, creatinine levels, and grading of retinopathy at the time of diagnosis of T2D and these variables were therefore not included in the analyses.

Since a large proportion of persons with T2D in Sweden reach HbA1c targets, indicating high overall quality of diabetes care compared with many other countries, similar challenges in terms of detecting persons with T2D at an early stage of hyperglycemia seem likely in other European countries and parts of the world. It also seems likely that slightly lowering glycemic targets (eg, from 52 mmol/mol to 48 mmol/mol) in patients with established T2D, often intensively debated, may have relatively little influence on prognosis,^16^ whereas many individuals with much higher levels remain undetected in turn leading to complications already at diagnosis. Therefore, we view early detection of T2D as a key challenge to resolve in the field of T2D.

In conclusion, intensified screening for T2D in clinical practice is needed in Sweden since almost one-fifth of these persons have retinopathy at diagnosis indicating long-standing hyperglycemia. The prevalence of DR was higher in certain patient groups including men, birthplace outside of Sweden, and those with a history of stroke, kidney, disease, and high SBP. Further research is needed to develop efficient strategies and programs to not only screen for T2D at random in clinical practice but also more structured screening to detect T2D earlier. This is of particular concern since many persons may have hyperglycemia before diagnosis and are not targets of efficient prevention strategies for complications before diagnosis.

## Supplementary material

10.1136/bmjdrc-2023-003976 online supplemental material 1

## Data Availability

Data are available upon reasonable request.
