# Supplementary material for: Prevalence and risk factors for diabetic retinopathy at diagnosis of type 2 diabetes: an observational study of 77 681 patients from the Swedish National Diabetes Registry
Source: BMJ Open Diabetes Res Care. 2024 Jun 4;12(3):e003976. doi: 10.1136/bmjdrc-2023-003976 (PMC11163631; doi:10.1136/bmjdrc-2023-003976)
Supplement: online supplemental material 1 [file bmjdrc-2023-003976supp001.docx]

**Supplementary appendix**

**Prevalence and risk factors for diabetic retinopathy at diagnosis of type 2 diabetes: an observational cohort study of 77681 patients from the Swedish National Diabetes Registry**

Sheyda Sofizadeh ^1,2^, Katarina Eeg-Olofsson ^2-4^, Marcus Lind ^1, 2, 4^

1. Department of medicine, NU-Hospital Group, Uddevalla, Sweden
2. Department of Molecular and clinical medicine, University of Gothenburg, Sweden
3. National Diabetes Register, Centre of Registers, Gothenburg, Sweden
4. Department of Medicine, Sahlgrenska University Hospital, Gothenburg, Sweden

# Table of contents

[ICD codes 3](#_Toc164715579)

[ATC-codes 4](#_Toc164715580)

[Table S1. Summary of missing data in main cohort 5](#_Toc164715581)

[Table S2. Patient characteristics for sub-cohort participants 6](#_Toc164715582)

# ICD codes

| Coronary heart disease | I20, I21, I22, I23, I24, I25 |
| --- | --- |
| Stroke | I61, I62, I63, I64 |
| Atrial fibrillation | I48 |
| Heart failure | I50 |
| Peripheral arterial disease | I702 |
| CABG | FNA0, FNA00, FNA10, FNA20, FNA96, FNB00, FNB20, FNB96, FNC10, FNC20, FNC30, FNC40, FNC50, FNC60, FNC96, FND10, FND20, FND96, FNE00, FNE10, FNE20, FNE96, FNF00, FNF10, FNF20, FNF30, FNF96 |
| Chronic Kidney Disease (CKD) | N181, N182, N183, N184, N189, N199 |

# ATC-codes

| Acetylsalycylic acid | B01AC06, N02BA01, N02BA51 |
| --- | --- |
| Lipid lowering drug | C10 |
| Type of hypertensive treatment | C02, C03, C07, C08, C09 |
| ACEi - Angiotensin converting enzyme inhibitor | C09A, C09B |
| ARB - Angiotensin receptor blocker | C09C, C09D |
| Alpha blocker | C02CA |
| Beta blocker | C07 |
| Calcium channel antagonist / blocker | C08C |
| Diuretics | C03A, C03B, C03C, C03D, C03E |

# Table S1. Summary of missing data in main cohort and excluded patients

|  | **Main cohort n=77 681** | | | | **Excluded  n=61 207** | | **Total**  **N = 138 888** | |
| --- | --- | --- | --- | --- | --- | --- | --- | --- |
|  | **N** | | | **%** | **N** | **%** | **N** | **%** |
| **Smoking** | |  | 17959 | 23.12 | 15778 | 25.78 | 33737 | 24.29 |
| **BMI** | |  | 19517 | 25.12 | 16701 | 27.29 | 36218 | 26.08 |
| **HbA1c at inclusion** | |  | 4321 | 5.56 | 3939 | 6.44 | 8260 | 5.95 |
| **Systolic blood pressure** | |  | 7413 | 9.54 | 8058 | 13.17 | 15471 | 11.14 |
| **Diastolic blood pressure** | |  | 7472 | 9.62 | 8127 | 13.28 | 15599 | 11.23 |

# Table S2. Patient characteristics for sub-cohort participants

| **Characteristics** | **Categories** | **Sub-cohort 1** | **Sub-cohort 2** | **Sub-cohort 3** | ***p*-value** | **SMD** |
| --- | --- | --- | --- | --- | --- | --- |
|  |  | N=73350 | N=56764 | N=52697 |  |  |
| Age years, mean (SD) |  | 62.62 (12.39) | 62.65 (12.28) | 62.66 (12.25) | 0.810 | 0.002 |
| Age category, n(%) | <55 years | 18437 (25.1) | 14118 (24.9) | 13019 (24.7) | 0.433 | 0.009 |
|  | 55-64 years | 19505 (26.6) | 15225 (26.8) | 14202 (27.0) |  |  |
|  | 65-74 years | 23254 (31.7) | 18100 (31.9) | 16865 (32.0) |  |  |
|  | 75+year | 12154 (16.6) | 9321 (16.4) | 8611 (16.3) |  |  |
| Sex female, n(%) |  | 30139 (41.1) | 23162 (40.8) | 21618 (41.0) | 0.569 | 0.004 |
| Smoking, n(%) | Smoking | - | - | 7938 (15.1) | - | - |
| HbA1c (mmol/mol) at inclusion, mean (SD) |  | 58.07 (20.93) | 57.57 (20.64) | 57.61 (20.71) | <0.001 | 0.016 |
| HbA1c (mmol/mol) at inclusion, n(%) | <48 | 26403 (36.0) | 21058 (37.1) | 19570 (37.1) | <0.001 | 0.020 |
|  | 48-52 | 15715 (21.4) | 12255 (21.6) | 11381 (21.6) |  |  |
|  | 53-57 | 7809 (10.6) | 5957 (10.5) | 5508 (10.5) |  |  |
|  | 58-70 | 9165 (12.5) | 6862 (12.1) | 6317 (12.0) |  |  |
|  | >70 | 14258 (19.4) | 10632 (18.7) | 9921 (18.8) |  |  |
| Place of birth, n(%) | Sweden | 56013 (76.4) | 43322 (76.3) | 40099 (76.1) | <0.001 | 0.007 |
|  | Europe except Sweden | 8490 (11.6) | 6625 (11.7) | 6206 (11.8) |  |  |
|  | North America | 184 ( 0.3) | 144 ( 0.3) | 134 ( 0.3) |  |  |
|  | South America | 625 ( 0.9) | 506 ( 0.9) | 474 ( 0.9) |  |  |
|  | Asia | 6175 (8.4) | 4730 (8.3) | 4439 (8.4) |  |  |
|  | Africa | 1851 (2.5) | 1428 (2.5) | 1338 (2.5) |  |  |
|  | Oceania | 12 (0.0) | 9 (0.0) | 7 (0.0) |  |  |
| Lipid lowering therapy, n(%) |  | 27495 (37.5) | 21510 (37.9) | 19901 (37.8) | 0.826 | 0.006 |
| Stroke, n(%) |  | 2737 (3.7) | 2057 (3.6) | 1900 (3.6) | <0.001 | 0.004 |
| Coronary heart disease, n(%) |  | 8719 (11.9) | 6804 (12.0) | 6273 (11.9) | 0.006 | 0.002 |
| Atrial fibrillation, n(%) |  | 5847 (8.0) | 4493 (7.9) | 4159 (7.9) | 0.287 | 0.002 |
| Heart failure, n(%) |  | 3343 (4.6) | 2561 (4.5) | 2329 (4.4) | 0.111 | 0.004 |
| Peripheral arterial disease, n(%) |  | 644 (0.9) | 484 (0.9) | 452 (0.9) | 0.005 | 0.002 |
| Coronary artery bypass graft, n(%) |  | 929 (1.3) | 772 (1.4) | 721 (1.4) | 0.027 | 0.006 |
| Chronic Kidney Disease, n(%) |  | 660 (0.9) | 520 (0.9) | 469 ( 0.9) | 0.001 | 0.002 |
| Any hypertensive treatment, n(%) |  | 47369 (64.6) | 37191 (65.5) | 34494 (65.5) | <0.001 | 0.013 |
| Acetylsalycylic acid, n(%) |  | 13621 (18.6) | 10616 (18.7) | 9815 (18.6) | 0.832 | 0.002 |
| Angiotensin converting enzyme inhibitor, n(%) |  | 17887 (24.4) | 14202 (25.0) | 13158 (25.0) | 0.012 | 0.010 |
| Angiotensin receptor blocker, n(%) |  | 18850 (25.7) | 14819 (26.1) | 13726 (26.0) | 0.189 | 0.006 |
| Alpha blocker, n(%) |  | 549 (0.7) | 443 (0.8) | 413 (0.8) | 0.722 | 0.003 |
| Beta blocker, n(%) |  | 24417 (33.3) | 19025 (33.5) | 17598 (33.4) | 0.689 | 0.003 |
| Calcium channel antagonist / blocker, n(%) |  | 18820 (25.7) | 14894 (26.2) | 13860 (26.3) | 0.014 | 0.010 |
| Diuretics, n(%) |  | 14776 (20.1) | 11588 (20.4) | 10739 (20.4) | 0.416 | 0.004 |
| Education category, n(%) | 9 years | 34896 (47.6) | 27014 (47.6) | 25062 (47.6) | 0.985 | 0.004 |
|  | 10-12 years | 20825 (28.4) | 16114 (28.4) | 14985 (28.4) |  |  |
|  | College/university | 16678 (22.7) | 12927 (22.8) | 11997 (22.8) |  |  |
| Systolic blood pressure (mmHg), mean (SD) |  | - | 137.5 (17.3) | 135.6 (16.20) | 0.963 | 0.001 |
| Systolic blood pressure (mmHg), n(%) | <110 | - | 1481 (2.6) | 1380 (2.6) | 0.995 | 0.003 |
|  | 110– < 120 | - | 4632 (8.2) | 4304 (8.2) |  |  |
|  | 120– < 130 | - | 11588 (20.4) | 10809 (20.5) |  |  |
|  | 130– < 140 | - | 15626 (27.5) | 14471 (27.5) |  |  |
|  | => 140 | - | 23437 (41.3) | 21733 (41.2) |  |  |
| Diastolic blood pressure (mmHg), mean (SD) |  | - | 81.2 (10.63) | 80.6 (10.2) | 0.959 | <0.001 |
| Diastolic blood pressure (mmHg), n(%) | < 60 | - | 509 (0.9) | 472 (0.9) | 1.000 | 0.001 |
|  | 60– < 70 | - | 4763 (8.4) | 4429 (8.4) |  |  |
|  | 70– < 80 | - | 15353 (27.0) | 14277 (27.1) |  |  |
|  | 80– < 85 | - | 16607 (29.3) | 15403 (29.2) |  |  |
|  | => 85 | - | 19532 (34.4) | 18116 (34.4) |  |  |
| BMI (kg/m^2^), mean (SD) |  | - | 30.9 (5.9) | 31.4 (5.9) | 0.690 | 0.002 |
| BMI (kg/m^2^), n(%) | <18.5 | - | 152 ( 0.3) | 141 ( 0.3) | 0.998 | 0.002 |
|  | 18.5-<25 | - | 6237 (11.0) | 5805 (11.0) |  |  |
|  | 25– < 30 | - | 19883 (35.0) | 18466 (35.0) |  |  |
|  | 30– < 35 | - | 17622 (31.0) | 16381 (31.1) |  |  |
|  | => 35 | - | 152 (0.3) | 141 (0.3) |  |  |

SD: Standard deviation (SD). SMD: Standardized mean difference. P-values are obtained from t-test for continuous variables and Chi^2^-tests for frequencies.
